# Why AMD is a disease of ageing and not of development: mechanisms and insights

**DOI:** 10.3389/fnagi.2014.00151

**Published:** 2014-07-10

**Authors:** Kaushal Sharma, Neel Kamal Sharma, Akshay Anand

**Affiliations:** ^1^Neuroscience Research Lab, Department of Neurology, Post Graduate Institute of Medical Education and ResearchChandigarh, India; ^2^Neurobiology-Neurodegeneration and Repair Laboratory, National Eye InstituteBethesda, MD, USA

**Keywords:** age related macular degeneration, metabolism, 7-ketocholesterol, angiogenic proteins, VEGF, degenerative diseases, complement factors, developmental disorders

## Abstract

Ageing disorders can be defined as the progressive and cumulative outcome of several defective cellular mechanisms as well as metabolic pathways, consequently resulting in degeneration. Environment plays an important role in its pathogenesis. In contrast, developmental disorders arise from inherited mutations and usually the role of environmental factors in development of disease is minimal. Age related macular degeneration (AMD) is one such retinal degenerative disorder which starts with the progression of age. Metabolism plays an important role in initiation of such diseases of ageing. Cholesterol metabolism and their oxidized products like 7-ketocholesterol have been shown to adversely impact retinal pigment epithelium (RPE) cells. These molecules can initiate mitochondrial apoptotic processes and also influence the complements factors and expression of angiogenic proteins like VEGF etc. In this review we highlight why and how AMD is an ageing disorder and not a developmental disease substantiated by disrupted cholesterol metabolism common to several age related diseases.

## Introduction

Age related macular degeneration (AMD) is described by irreversible vision loss in older age. The disease pathology emerges with the degeneration of macula which forms the central part of retina. The macula consists of photoreceptor (rods and cones) important for central vision. As AMD symptoms appear, characteristic features such as formation of drusen, consisting of active and inactive complement associated inflammatory products, aggregate of lipoprotein, cell debris, oxysterols, oxidized phospholipids and *Alu* RNA deposits begin to emerge later in life and not during development. These aggregates deposit in the extra-cellular space between Bruch’s membrane and retinal pigment epithelium cells (RPE). Gradual and consistent effects of these aggregates gradually cause degeneration of these cells followed by global atrophy of RPE cells, commonly known as geographic atrophy (GA). Besides, active inflammatory components of these deposits between Bruch’s membrane and RPE, stimulate angiogenic factors (e.g., VEGF, TGFB etc.) which act on choriocapillary network beneath the Bruch’s membrane and stimulate proliferation to new blood vessels (a process called neovascularization). These newly formed blood vessels can outgrow into the RPE cells and result in disruption of RPE cell integrity and function which is well preserved in early life. Understanding the complexity of mechanisms through genetics, epigenetics, metabolic pathways and risk factors have provided insight about the participation of cellular pathways that resemble aging, but not early or late development. Cells that lose their capacity to divide by a phenomenon called cell senescence undergo ageing. Several impaired cellular processes could lead to cellular and morphological changes in the cell over time in association with environmental factors in complex manner ultimately resulting in ageing. These cellular processes include: metabolic pathways (Uchiki et al., 2012), telomere shortening, impaired mechanism of autophagy, disrupted proteolytic and lysosomal function (Viiri et al., 2013); decline in ability to combat oxidative stress (Cutler et al., 2004) and enhanced mitochondrial dysfunction etc. all of which can disrupt homeostasis of the cell. Therefore, age related changes in the cell are the basis of several diseases which are termed as age related diseases. Hence, the age related diseases depend on the degree of ageing in cells. Several genetic loci have been postulated to drive age related changes in the organism even in pre-mature age (Friedman and Johnson, 1988; Kennedy et al., 1995; Hernandez et al., 2010). Therefore, such impaired cellular, genetic or molecular processes coupled with environmental changes result in age related disorders. Instead of age related disorders, the developmental disorders are mostly inherited when other cellular processes are intact. Infact the environmental factors also play an important role in developmental disorders but not in a manner representative of diseases of ageing. Therefore, future effective interventions to combat diseases of aging shall require comprehensive understanding of gene regulatory networks than single gene replacement strategies.

### Interaction between environment factors and genetic loci

**Figure 1:**
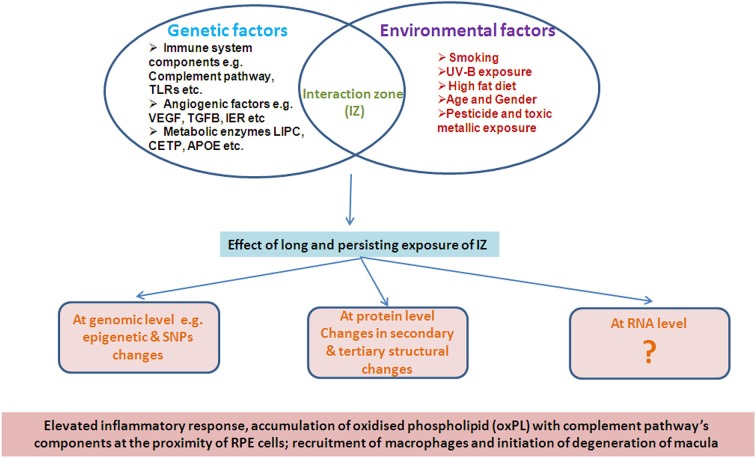
**Illustrative representation of interaction of Genetic and environment factors**.

It is evident that the disease pathology of AMD is equally influenced by both environmental as well as genetic factors in a complex manner such that the effects are not manifest early in life. Several environmental factors such as age, sex, diet, smoking and demographic distribution have been reported to be strongly associated with AMD pathology (Figure 1), unlike a developmental disorder.

### Epigenetic changes introduced by environmental factors in AMD

The epigenetic changes in the genome have been well defined in several genetic diseases. These epigenetic alterations affect the 5 expression level of several important genes apart from exerting protective role on host genome by either preventing activation of restriction enzymes or other mechanisms. Several cancers are believed to progress through similar chemical modifications (epigenetic changes) of the genome which impact the expression pattern of regulatory genes. The fundamental chemical changes include methylation, phosphorylation, acetylation, *etc*. which are reportedly involved in eukaryotes. Several environmental factors like smoking, stress, dietary habits etc. have been well described in introducing chemical changes on the genome (Lim and Song, 2012). Ageing can also influence the methylation pattern of the genome (Bollati et al., 2009). These studies suggest that apart from the inherited epigenetic changes, these changes can also be introduced by several modulatory environmental factors especially smoking and deficiency of anti-oxidants in diets in a later stage of life than at the developmental stage.

Similarly, there are several co-morbid conditions which have been found to be associated with manifestation of AMD. Persistent hypertension, heart disease and diabetes, accelerate AMD pathogenesis. Lately, it has also been revealed that Alzheimer’s disease appears to have concurrence with AMD pathology suggesting the role of several common gene loci indicating that their associated pathological mechanisms, underlying cascade of downstream events, converge to cause the two diseases of ageing. This review highlights the role of various candidate genes that play an independent role in progression of AMD and digresses on spectrum of gene association studies which modify AMD pathology in a manner not characteristic of a development disorder.

The genes associated with AMD can be conveniently categorized into following groups based on their direct or indirect association reported in various studies:

1. Complement system components: e.g., complement factor H (CFH), component C2, component C3 and complement factor I (CFI). Moreover, TLRs receptor components of innate immunity have also been found to be strongly associated with AMD pathogenesis. Therefore, in general the components of innate immunity have predominant role in progression of AMD.
2. Enzymes involved in metabolic processes: Hepatic Lipase (LIPC), cholesteryl ester transfer protein (CEPT) and Apolipoprotein E (APOE) are involved in different stages of lipid metabolism.
3. Angiogenic factors: VEGF has been reported as the strongest angiogenic factor. Several angiogenic factors which have been reported till now are either associated with AMD or are reported to be associated with degenerative and inflammatory diseases. e.g., VEGF, COL genes, TGFBR1, immediate early response (IER3) etc.
4. Proteases: Several proteases have been implicated in AMD pathophysiology including degradation of extracellular matrix. For example, Tissue inhibitor metalloprotenases 3 (TIPM3), IER3 and numerous multifunctional proteins which have several cellular functions like ADMS2, HTRA1 *etc*. play a non redundant role in pathologies of AMD.
5. Proteins in apoptosis and cellular regulation: e.g., *TNFRSF10A* and regulatory cellular enzymes like*DICER*.

AMD literature is replete with both confirmatory and conflicting reports of several genes and their polymorphisms which have been found to be associated with AMD.

## Proteins involves in metabolic processes

Metabolic processes constitute the normal biochemical pathways in the cell which are involved in transportation and biosynthesis of biomolecules including several regulatory processes of the cells. AMD can be regarded as a disease caused by disruption or abnormal metabolic processes especially lipid metabolism. The metabolic processes which are associated with lipid metabolism are one of the major factors in AMD pathogenesis. Several enzymes which participate in lipid and lipid associated metabolism have been found to be associated with initiation and progression of AMD pathology. Several population based genetic and animal based studies have revealed the role of proteins involved in lipid metabolism and their associated receptors in AMD. Important enzymes which participate in lipid metabolism, including human hepatic lipase gene (LIPC), lipoprotein lipase gene (LPL), cholesterol ester transferase gene (*CEPT*) and ABC binding cassettes A1 (*ABCA1*) gene have been found to be associated with AMD pathology. Lipoprotein lipase acts when systemic cholesterol levels fall along with the function of *ABCA1* leading to formation of* APOE*, *APOA1* and HDL. When cholesterol levels rise, the action of CEPT comes into play which removes the cholesterol ester (CE) from HDL and convert into LDL by enzymatic process. LIPC and LPL both have dual functions i.e., as triglyceride hydrolase as well as in receptor mediated endocytosis of cholesterol at different stages of metabolism.

Recent studies have demonstrated composition and sub-structures of drusen comprising of 3.2% dry weight of long fatty acid esterified and non-esterified cholesterol and about 30 different types of proteins, including apolipoproteins (apo E and apo B) (Burns and Feeney-Burns, 1980; Pauleikhoff et al., 1992; Crabb et al., 2002; Li et al., 2005). Lipid metabolizing enzyme human Lipase C (*LIPC*) is expressed in hepatic cells and adrenal gland whose principal function is to convert intermediate-density lipoprotein (IDL) to low-density lipoprotein (LDL) which have been widely investigated in AMD. The lipids and lipoprotein contents of drusen between Bruch’s membrane and RPE are basically distinct from the plasma contents and are comprised of free and esterified cholesterol, phosphotidylcholine (PC), apolipoproteins B-100, A-I and ApoE (Curcio et al., 2001; Wang et al., 2010). Several genetic studies have investigated the association of LIPC with AMD pathogenesis. Recently, Neale et al. (2010) implicated the association between AMD and a variant in the hepatic lipase gene (*LIPC*) (rs493258). They found *LIPC* association was strongest for a functional promoter variant, rs10468017, that influences *LIPC* expression and serum HDL levels with a protective effect of the minor T allele (HDL increasing) for advanced wet and dry AMD.

ApoE, another protein, also involved in lipid transportation and metabolism has also been extensively investigated in AMD pathology. ApoE is a polymorphic protein which plays multiple roles in lipid homeostasis in central nervous system and metabolism of plasma lipid (Mahley, 1988). APOE gene has three different variants called APOE2, APOE3 and APOE4 which are due to the variation in two SNPs in the APOE gene, rs429358 and rs7412.Immunoreactivity of APOE in the eye has been restricted to Bruch’s Membrane, Müller cells, RPE, drusen and basal deposits (Klaver et al., 1998; Anderson et al., 2001; Malek et al., 2003). mRNAs for APOE are synthesized by the RPE and by Muller cells in the neural retina (Anderson et al., 2001). Polymorphism in APOE gene is a risk factor for various neurodegenerative diseases, and the protein has been shown in the lesions of these disorders. APOE gene is also known to play a major role in AMD pathology. Some association studies have reported that APOE4 variant provides some protection from developing AMD. APOE2 variant is more common in individuals with AMD and may play a role in its progression (Klaver et al., 1998; Souied et al., 1998; Schmidt et al., 2002; Baird et al., 2004). These results are in variance to the animal studies in whichAMD severity has been found to be associated with ApoE4 expressing aged mice which were subjected to high fat cholesterol. Even the major complex diseases such as atherosclerosis, Alzheimer’s disease, and stroke, are characterized by strong association betweenAPOE4allele and disease (Kalaria, 1997; Weller and Nicoll, 2003; Enzinger et al., 2004).

Several ApoE knockout mice studies have indicated the role of ApoE affecting systemic cholesterol levels (Plump et al., 1992; Zhang et al., 1992). Dithmar et al. have shown ultrastructural changes of the Bruch’s membrane in ApoE deficient mice. They have found elevated fraction of membrane bound electron-lucent particle accumulation between two membranes in ApoE −/− mice as compared to control mice (Dithmar et al., 2000). Lately, it has been found that AMD pathology, like sub-retinal drusen deposition, drusenoid, thickening of BM, atrophy, hypo and hyper-pigmentation of RPE are dependent on ApoE isoforms. AMD severity has been found to be associated with ApoE4 over expressing senescent mice which were subjected to high fat cholesterol (Nguyen-Legros and Hicks, 2000). Moreover, cell-membrane remodeling is an essential mechanism for maintenance of retina and its normal functioning (Klaver et al., 1998), which may be regulated by APOE. Protective function of PAOE4 are believed to arise from its inability to form dimers as compared to APOE2 and APOE3 variants which may permit easier transportation of lipids through Bruch’s membrane (Souied et al., 1998). Lipids accumulate with the advancement of age, which may lead to the creation of hydrophobic barrier in the Bruch’s membrane causing AMD.

On the other hand, the involvement of APOE has also been widely explored in AD. It has been reported that severe oxidative changes in protein and lipid have been shown in synaptosome of APOE knockout mice after Aβ induced insult (Lauderback et al., 2001). However, it has also been hypothesized that APOE may function as “pathological chaperon” in stabilizing the Aβ sheet structure and can also alter the normal Aβ in plaque (Ji et al., 2001). Moreover, it has also been investigated that the burden of amyloid-β-peptide was diminished in APP^*V717F*+/–^ Apoe−/− as compared to APP^*V717F*+/–^ Apoe^+/–^ and Apoe−/− triglycerides (TG) mice in 20 month aged mice. The burden of Aβ in the hippocampal region was higher in Apoe+/+ transgenic mice as compared to Apoe+/− and Apoe−/− mice. This suggests that even though both pathologies implicated similar elements but the final effect are quite opposite (Bales et al., 1999). Therefore, these studies have exemplified the role of APOE in two major age related degenerative disease, suggesting that APOE may have the dual role in both AMD and AD.

It was also discovered that age and high fat cholesterol diet alone were not sufficient factors to stimulate AMD pathology apart from isoform dependent pathology. In experimental mice, advanced features of AMD i.e., neovascularization, with the presence of VEGF in drusen, has been found to be deposited in BM region, which was also reported earlier, implying the role of ApoE function in both types of AMD. The components responsible for dry AMD further stimulate genetic cascade which leads to activation of angiogenic molecules or metalloproteases (Malek et al., 2005). It is argued that these changes are not critical to developmental and the disorders of developmental. Rudolf et al. (2005) have further distinguished the role of lipid metabolism in stimulation of angiogenic factor (VEGF) in LDL-receptor knockout mice. Interestingly, it has been shown that in LDL- knockout mice, the expression of VEGF was high as compared to controls.

Another lipid metabolizing enzymes cholestryl ester transfer protein (CEPT) is a plasma protein that plays an important role in cholesterol metabolism by facilitating the transportation of triglyceride and cholesteryl ester from VLDL to HDL or vice versa. Several genetic studies have demonstrated conflicting results of CEPT association with AMD progression. Recently, Yu et al. (2011) reported no association between CEPT polymorphism and AMD pathology but demonstrated that other cholesterol metabolizing enzymes like LIPC and a cholesterol transporter protein *ABCA1* may have role in AMD pathophysiology such that the cumulative effect gets manifest in ageing and not earlier in life.

Several cholesterol metabolic intermediates have been found to stimulate pathological mechanisms involved in AMD like angiogenesis, inflammation etc. 7-ketocholesterol (7-Kch), one of the oxidized component of cholesterol metabolism, has been found to stimulate angiogenic factors and inflammatory processes (Ignacio and Larrayoz, 2010). Oxidation of cholesterol into 7-Kch is carried out by both enzymatic and non-enzymatic processes. Non-enzymatic processes are carried out by the involvement of singlet oxygen species and free radical mechanism. The mechanism mediated by singlet oxygen species requires photosensitizer (Thomas et al., 1987) and free radical based mechanism carried out by the involvement of metal catalysts like Cu^+2^/Fe^+2^ (Dzeletovic et al., 1995; Brown et al., 1996). These studies have also suggested the probable role of Fe^+2^ and oxidation mediated processes in AMD pathophysiology. Currently, only free radicals based mechanisms have been found to be occur in retina, however, no evidence for singlet mediated cholesterol oxidation into 7-Kch has been reported.

Most important enzymatic oxidation of cholesterol takes place with the involvement of CYT46A1 and CYP27A1 loci of cytochrome P450 family which forms 24-hydroxycholesteorl (24-Hch) and 27-hydroxycolesterol (27-Hch), respectively (Björkhem et al., 2009). The high levels of 27-Kch have been reported in macrophages foam cells and in atherosclerotic plaque, but has not been documented in retina (Björkhem et al., 1994; Luoma, 2008). These population based genetic studies coupled with animal experiments indicate a prominent role of cholesterol metabolism in AMD pathology indicating the longitudinal and cumulative effects that do not alter phenotype earlier in life. Recently, it has been demonstrated that the metabolic components of lipid metabolism influence and activate the angiogenic factors, complement factors and other regulatory components by disrupting their regulatory pathways not critical in early life.

## Effects of cholesterol metabolism intermediates on complement factors

Data from the previous research strongly indicates that the dysregulation of innate innunity can induce and result in progression of AMD. Several GWAS and rare variants analysis studies have strongly specified the role of alternative complement pathway in AMD (Richards et al., 2007; Anderson et al., 2010; Raychaudhuri et al., 2011; Khandhadia et al., 2012; Tuo et al., 2012) and it has been well established that the dysregulation of complement pathways, especially alternative complement pathway is responsible for AMD pathophysiology and aberrant regulation of these factors may lead to progression of AMD.

Recently, several studies have demonstrated the effects of cholesterol metabolic components which influence these intermediate components like in case of atherosclerosis. It has been already established by Hasselbacher et al. in 1980 that cholesterol has high potential to activate the alternative complement pathways componetnts which have been demontrated by immunoelectrophoretic reactivity of C3 and properdin factor B (Hasselbacher and Hahn, 1980), however none of the the complement factors was activated when liposomes, containing sphingomyelin as a phospholipid (Alving et al., 1977), was used. Therefore, these studies have suggested the potetial role of cholesterol intermediates and alternative complement pathway in influencing disease outcome. The mechanism behind cholesterol dependent complement activation has been illustrated and found to be associated with C5/C3 conversion into cholesterol crystals which consequently induce alternative complement cascades which may be stabilized by factor B and D. It has also been examined that factor I (inactivator of factor C) shows affinity with cholesterol crystals thus indicating cholesterol mediated complement pathway activation proceeds by transformation activity not by the involvement of factor C (Vogt et al., 1985). Consistent with these studies the generation of anaphylatoxins and C5b-9 complex has also been observed and suggested to play a role in the inflammatory processes mediated by cholesterol and its intermediators, as seen in artherosclorosis, AMD and Alzheimers disease (Seifert and Kazatchkine, 1987). The accumulation of lipofuscin, with several oxysterol, have been shown to have amplified CFH activity along with increased C3b suggesting lipid intermediate based complement dysregulation. This may lead to features reminscent of AMD pathology (Sparrow et al., 2012). Recently, it has been further reported that the CFH interaction with oxidized products of cholesterol can modulate the protective effect of CFH. The protective allele of CFH in AMD rs1061170 has been shown to exert strong affinity and inhibitory effect of CFH upon binding with oxidized phospholipid mediated by changes in the expression of genes responsible for neovascularization, inflammation and macrophages infiltration. This study has suggested the interaction of oxidized phospholipid could bring changes in the protective role of CFH by modulating other genes involving various processes associated with AMD pathology (Shawa et al., 2012). Additionally, Sharma et al. have also shown the reduced levels of CFH in AMD patients (Sharma et al., 2013b). Similarly the same group, also reported altered CCL-2 levels in AMD patients (Anand et al., 2012). Both results suggest that impaired macrophages recruitment in AMD pathology.

Apart from interaction and effects on complement factors, lipid metabolic intermediates have also accounted for their direct consequences on inflammatory responses by affecting gene regulation involved in such processes. Through *in vitro* studies by Shawa et al. (2012) it has been demonstrated that the interaction of oxidized phospholipids could upregulate the expression of CCR-2, IL-6, IL-8, CD-36 and VEGF. The increased expression of CCR-2 suggests augmented infiltration of macrophages at the site of accumulated oxidized phopholipids which further influence the intergrity of RPE cells by modulating the expression levels of proinflammatory, inflammatory and angiogenic factors through NFkB pathway. Recently, Amaral et al. have shown in a rat model that the administration of 7-Kch which induces the macrophage infiltration at site of accumulation, have displayed increased immunolocalization of CD68 (Amaral et al., 2013). This could result in enhanced expression of proinflammatory genes along with angiogenic factors in moncytic THP-1 cells (Erridge et al., 2007). Additionally, several *in vitro* studies have also shown that accumulated intermediates of lipid and cholesterol metabolism can induce the endoplasmic reticulum (ER) stress (Lee et al., 2009; Huang et al., 2012) by involvement of various NFkB pathways (Dugas et al., 2010; Larrayoz et al., 2010; Huang et al., 2012).

The role of macrophages in atherosclerosis against oxysterols have also been extensively investigated. These studies suggest that the regulation of synthesis of inflammatory factors like TNF-α, IL-8, IL-6 etc. is mediated by MEK/ERK and AP-1 processes (Lemaire-Ewing et al., 2005, 2009; Erridge et al., 2007). The inhibition of these proinflammatory factors induced by some oxysterols could be regulated via liver X receptor (LXR) possessing the capacity to suppress their synthesis at the time of atherosclerosis (Calkin and Tontonoz, 2010; Im and Osborne, 2011). Collectively, these studies indicate that macrophages mediated proinflammatory processes induced by accumulated lipid/cholesterols intermediates play a major role in diseases like Alzheimer’s disease, Atherosclerosis and AMD all of which occur with ageing. Lately, an elaborate role of cholesterol in macrophages have been illustrated by participation of lipid metablic processes in AMD. Sene et al. have demonstrated that impaired cholesterol transport in macrophages could influence the macrophages polarization into particular cell type consequently impacting the pathological hallmarks in AMD (Sene et al., 2013).

Conclusively, these studies have suggetsed the importance of lipid/cholesterol metabolites in inducing the AMD pathological hallmarks by several regulatory components of innate immunity.

These studies demontrate that lipids/cholesterol metabolites have their impact on regulation of complement factors, interaction with protective variant of CFH and its modulation, macrophage infiltration, and regulation of proinflammatory/inflammatory genes suggesting the role of cholesterol metabolism and its metabolites on the complement pathway. These studies also suggest that cholesterol transport influences the role of macrophages in progression of AMD such that these effects do no operate early in life.

## Cholesterol metabolites and angiogenic factors

Lipids and choleserols are essential for biological functions ranging from membrane trafficking to signal transduction and can activate various important regulatory molecules which could be associated with several pathological hallmarks of different types of diseases. Several studies have increased our understanding of direct relation of lipids and cholesterol intermediates in pathogenesis of inflammatory diseases and diseases affected by lipids deposition, at the molecular and cellular level. Neovascularization is one of the common features which affects about 10% of total AMD and can be influenced by several intermediates of cholesterol metabolism. Both angiogenic factors as well as matrix metalloproteases (MMP) can contribute in the progression of pathophysiology of choroidal neovascularization (CNV) in AMD. VEGF expression, expression of proangiogenic factors, and activation of metalloproteases are believed to play an important role in the formation of CNV in AMD. It is well understood that the biologic activities of VEGF-A, proangiogenic molecules like (TGFB, TIMP, IER etc.) and proinflammatory cytokines can be influenced by the interaction of cells with the extracellular matrix (ECM). Cell-ECM interaction, useful for integrity and functionality of cell, are influenced by the expression of these factors (VEGF, angiogenic and proangiogenic factors). ECM protein channel-ECM communications influence tissue remodeling events, including angiogenesis. Several angiogenic factors have been widely studied as one of the cardial feature of AMD pathophysiology: “neovascularization”. The neovascularization being the consequence of dysregulated action of genes involved in proangiogenesis, angiogenesis, extracellular proteins, and metalloproteases is the central thesis in wet AMD. These effects are additive, rarely affecting development milestones. *VEGF*, one of the potent angiogenic factor known, plays an important role in new vessels formation because it is involved in vascular development and have been strongly implicated and reported in the pathogenesis of AMD (Carneiro et al., 2012) as well as corneal neovascularization (Philipp et al., 2000). The *VEGF* family mainly binds with three types of *VEGF*Rs which include: *VEGF*R1, *VEGF*R2, and *VEGF*R3, as well as to co-receptors [such as heparan sulphate proteoglycans and neurophilin] (Hiratsuka et al., 1998). It has been investigated that the expression of VEGF can be influenced by components of complement pathway. When RPE cell culture were exposed with C3a/C5a, also present in drusen, it can significantly increase the expression of VEGF (Nozaki et al., 2006). Recently, we also reported VEGFR2 in AMD patients which are found to be significantly associated with disease pathology (Sharma et al., 2012a).

Similarly, *TGFBR1* also possesses the property to exert proangiogenic effects with its encoded protein forming a heteromeric complex with type II *TGF*-beta receptors and transducing the TGF-beta signal from the cell surface to the cytoplasm when bound to TGF-beta. This protein is a serine/threonine protein kinase. This gene is expressed in all tissues but more abundantly expressed in brain and heart. This protein is involved in several biological processes like induction of apoptosis, response to hypoxia, epithelial to mesenchymal transition, artery morphogenesis, signal transduction, regulation of transcription, negative regulation of endothelial cell differentiation, positive regulation of cell proliferation, collagen fibril organization *etc*.

Apart from above mentioned angiogenic genes, *IER3* has been postulated to be responsible for regulating death receptor mediated apoptosis, interaction with NF-κB pathways, and upregulated at irradiation, ionizing radiation, viral infection and at the time of other cellular responses. Numerous functional data relying on cell culture based studies and knock-out mouse models has revealed the involvement of *IER3* expression in immune functions and in the physiology of the cardiovascular system. Arlt et al. showed that deficiency of *IER3* expression in mice results in an aberrant immune regulation and enhanced inflammation, hypertension or impaired genomic stability (Arlt and Schäfer, 2011). Another report has documented the genetic network in injured retina reporting increased expression of *Ier3* gene with other transcription factors such as *Crem*, *Egr1, Fos, Fosl1, Junb, Btg2, Atf3*, and *Nr4a1* etc. Together, these genes implicate an angiogenic pathway attributed to *CFH* independent mechanism, popularly accepted to be responsible for almost half of AMD cases. None of these gene loci or their networks have been reported to be involved in a development disorder. Sasada et al. has also reported the effect of *IER3* which interferes with certain signaling pathways specifically NF-kB, MAPK/ERK and PI3K/Akt and have found aberrant immune function and increased inflammation, with an alteration of blood pressure and impaired in genomic stability (Sasada et al., 2008). Numerous patients based studies have also indicated a role of *IER3* in tumorigenesis. In pancreatic cancer patients, significant negative correlations have been observed between the *IER3* expression and serosal or arterial invasion of tumors. Thus, a positive *IER3* expression in tumor tissues may be associated with a better prognosis in pancreatic cancer (Sasada et al., 2008).

Several studies have been carried out to examine the effect of deposited oxidized sterols and 7-Kch on the expression pattern of these angiogenic proteins like VEGF. The effect of oxidized cholesterol and 7-Kch has been widely investigated in both animal as well as *in vitro* studies being responsible for inflammtory processes at accumulation site. Moreover, recent studies have validated the reports that phospholipidosis could increase several times in retina when exposed with these cholesterol intermediates (Miguet-Alfonsi et al., 2002; Vejux et al., 2008). Even though the activation of complement factor can also alter the expression of metalloproteases 2/9 as well as their activity along with imbalance in VEGF to PEDF ratio, these results suggest the effect of activated complement which could further induce the proangiogenic environment for neovascularization in AMD (Bandyopadhyay and Rohrer, 2012). Therefore, the activated complement components with oxidized sterols and 7-Kch can directly influence the expression of angiogenic and proangiogenic molecules.

Knockout mice for ApoE and the mice feeding high fat diet have shown several AMD like characteristics features. Increased implicit time and reduced oscillation potential amplitude were observed in these mice. Moreover, the scanning electron microscopic examination has also revealed that thickening of Bruch’s membrane and disorganized elastic lamina which results in stimulation of choroidal angiogenesis beneath the Bruch’s membrane. Therefore, the such studies suggest the role of *ApoE* and cholesterol which may provoke the nearby environment of RPE, Bruch’s membrane and molecules involved in adhesion of RPE and lamina by stimulating the angiogenic factors and matrix proteases (Ong et al., 2001).

**Figure 2:**
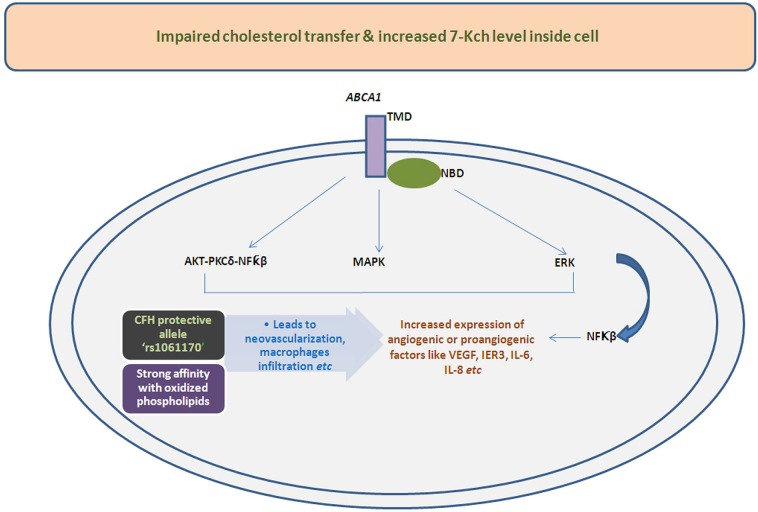
**Illustrative mechanistic flow chart functions of various signaling pathways leads to altered expression of angiogenic/proangiogenic factors influence by impaired transport and deposition of cholesterol or oxidized cholesterols due to hampered action of *ABCA1* transporter**. The deposited oxidized cholesterols components also have shown higher affinity with protective allele of CFH i.e., rs1061170 which can also changes the expression of angiogenic molecules.

Recently, the mechanistic view of activation of proangiogenic/angiogenic factors by oxidized sterols, 7-Kch or by impaired cholesterol transfer has been demonstrated by several investigators (Figure 2). Sene et al. (2013) had discovered the existence of impaired cholesterol transport in macrophages affecting ATP binding cassette A1 (ABCA1) transporter converting the naive macrophages into senescent macrophages. The effect of cholesterol accumulation inside macrophages has been found to be abnormal polarization of macrophages into two populations. The macrophage M1 only secretes proinflammatory and inflammatory components but polarization of M1 to M2 could pilot the secretion of angiogenic factor like VEGF, IL-8 *etc*. Therefore, these studies together signify the importance of cholesterol metabolism in AMD which is altered in neovascularization of choroid blood vessels.

The immediate effects of these deposited oxidized cholesterol components have also been examined in higher vertebrate i.e., monkey *etc*. The cholesterol intermediates 7-Kch have also shown the capacity to induce VEGF in monkeys. Moreira et al. have attempted to figure out the localization of 7-Kch in retina and examine the impending consequences by deposition of these oxidized cholesterol products in higher primates. The immunolocalization studies define the location of 7-Kch which was found to be deposited in between of choriocapillaries and Bruch’s membrane besides the surface of vascular endothelial cells. This was found to be 4–5 times higher when compared to control monkey retina. Further, *in vitr*o studies have demonstrated the induction of VEGF in ARPE-19 cells is 8–10 folds higher in 7-Kch induced retina as compared to those without induced cells. Moreover, they also found that the expression of VEGF was decreased when inhibitor for LXR receptor was exposed with anatagonist, however, no change in expression was noticed when activity of NFkB was inhibited, except IL-8. The study implies the role of 7-Kch in CNV induced by VEGF may be partially regulated by LXR receptor but independent on HIF-1 α and NFkB (Moreira et al., 2009).

## Apoptosis mediated by oxidized cholesterol and 7-Kch

**Figure 3:**
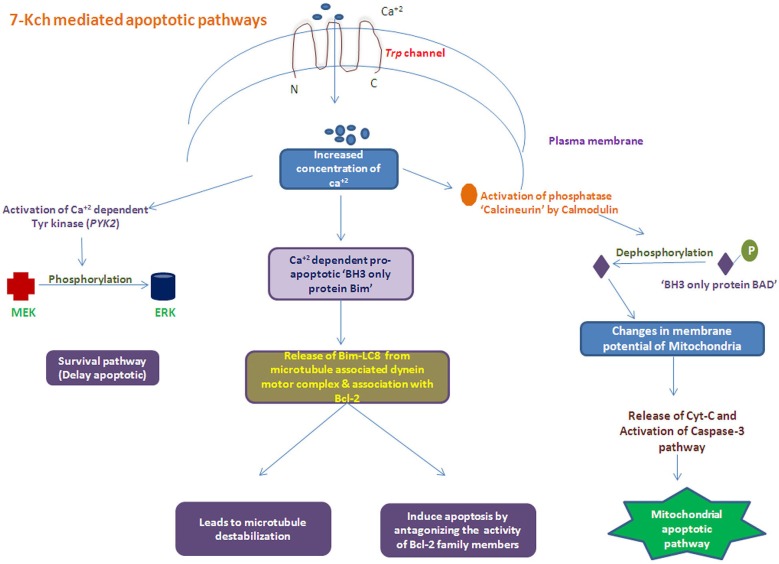
**Schematic of apoptosis process Induced with accumulated oxidized cholesterols (i.e., 7-Kch) by activating several apoptotic pathways**.

The 7-Kch exerts toxic effects by inflammation (Vejux et al., 2008; Larrayoz et al., 2010) and subsequently induce apoptosis of RPE cells mediated by mitochondrial apoptotic pathway (Miguet-Alfonsi et al., 2002; Ignacio and Larrayoz, 2010). Accumulated oxidized sterols, 7-Kch and impaired cholesterol transport leads to increase the level of Ca^+2^ inside cell mediated by *Trp* channel. This increased Ca^+2^ levels can activate the two types of apoptotic mechanisms: (i) dephosphorylation of “BH3 only protein Bad” which leads to mitochndrial apoptotic pathway; and (ii) activation of “pro-apoptotic BH3 only protein Bim” ultimately inducing apoptosis by inhibiting the activity of Bcl-2 family members proteins along with microtubultes destabilization (Figure 3). Recently, we have reported the reduced DcR1 (TNF-related apoptosis-inducing-ligand receptor-3) levels in AMD patients (Anand et al., 2014) where the angiogenic factors like CCR-3, VEGFR2, and eotaxin-2 (Sharma et al., 2012a,b, 2013a) were found to be increased in serum of these patients. These reults suggested the role of angiogenic factors with the involvement of apoptotic molecules may contribute to the disease pathology in these patients.

## Conclusion

Several age related disorders like AD, Parkinson’s disease (PD), atherosclerosis and AMD can be distinguished by several pathological hallmarks that appear at the time of ageing. Collectively, these studies suggest the imperative role of metabolic processes and various oxidized products which exert their additive effect on both complement and proangiogenic/angiogenic molecules with time. Several such reports indicate that altered cholesterol metabolism leads to pathological changes which results in accumulation of sterols, APOE, cholesterol oxidase and oxidized cholesterols in these diseases of ageing (Kannel et al., 1971; Martins et al., 2006; Moreira et al., 2009). The altered levels of LDL, HDL, ApoAI, ApoB and TG in AMD pathology is thus not a coincidence as these are rarely reported in developmental disorders (Nowak et al., 2005; Reynolds et al., 2010; Davari et al., 2013).

In this context, the generality of need to compare the critical mechanisms distinguishing an ageing disorder such as AMD from mechanisms akin to developmental disorders can provide insight for future interventions. Understanding the molecular mechanisms that prevent AMD from being a developmental disorder may therefore lead to effective therapies in future. For this, longitudinal comparative studies between developmental and degenerative disorders are warranted.

## Author’s contributions

Writing of manuscript: Kaushal Sharma, Akshay Anand, Neel Kamal Sharma. Editing of manuscript: Akshay Anand. Concept of the manuscript: Akshay Anand.

## Conflict of interest statement

The authors declare that the research was conducted in the absence of any commercial or financial relationships that could be construed as a potential conflict of interest.
